# Neuronal Nicotinic Acetylcholine Receptor Modulators Reduce Sugar Intake

**DOI:** 10.1371/journal.pone.0150270

**Published:** 2016-03-30

**Authors:** Masroor Shariff, Maryka Quik, Joan Holgate, Michael Morgan, Omkar L. Patkar, Vincent Tam, Arnauld Belmer, Selena E. Bartlett

**Affiliations:** 1 Institute of Health and Biomedical Innovation, Queensland University of Technology at Translational Research Institute, Brisbane, Australia; 2 Centre for Health Sciences, Bioscience Division, SRI International, Menlo Park, California, United States of America; 3 School of Psychology and Counselling, Queensland University of Technology, Brisbane, Australia; University of Leicester, UNITED KINGDOM

## Abstract

Excess sugar consumption has been shown to contribute directly to weight gain, thus contributing to the growing worldwide obesity epidemic. Interestingly, increased sugar consumption has been shown to repeatedly elevate dopamine levels in the nucleus accumbens (NAc), in the mesolimbic reward pathway of the brain similar to many drugs of abuse. We report that varenicline, an FDA-approved nicotinic acetylcholine receptor (nAChR) partial agonist that modulates dopamine in the mesolimbic reward pathway of the brain, significantly reduces sucrose consumption, especially in a long-term consumption paradigm. Similar results were observed with other nAChR drugs, namely mecamylamine and cytisine. Furthermore, we show that long-term sucrose consumption increases α4β2 * and decreases α6β2* nAChRs in the nucleus accumbens, a key brain region associated with reward. Taken together, our results suggest that nAChR drugs such as varenicline may represent a novel treatment strategy for reducing sugar consumption.

## 1. Introduction

Excess sugar consumption is implicated as one of the essential and underlying components of the current obesity epidemic, which is now a worldwide phenomenon [1, 2]. Indeed, binge sucrose drinking has been shown to repeatedly elevate dopamine levels in the nucleus accumbens (NAc) [3–6], a key feature of drugs of abuse [7–14]. Moreover, chronic intermittent sugar intake causes an increase in the expression of dopamine D1 receptors in the NAc, decrease in the expression of D2 receptors in the NAc and striatum [15–17] and also an increase in dopamine D3 receptor mRNA in the NAc and the caudate-putamen. Similar changes are noted in response to cocaine and morphine [18–24]. Furthermore, a decrease in enkephalin mRNA levels in the NAc[25] has been observed following intermittent sugar consumption [17], with similar observations in response to repeated injections of morphine [22, 23] or in cocaine-dependent human subjects [26]. Finally, during withdrawal from chronic sucrose exposure, rats show an imbalance in dopamine and acetylcholine, i.e. dopamine levels decrease whilst acetylcholine levels increase [27], similar to changes observed with several drugs of abuse, including morphine, nicotine and alcohol [28–30]. This adds impetus to investigate the limbic system as a possible therapeutic target to reduce sugar consumption.

The limbic system is an interconnected collection of brain structures including the NAc and the ventral tegmental area (VTA) that encode emotional states such as anticipation of reward and motivation [31]. In relation to sugar consumption, the mesolimbic system has been shown to display an exaggerated incentive salience response to cues for sucrose [32–34]. Indeed, animal studies have shown that long-term consumption of palatable food can cause changes in the brain reward pathways, suggestive of an imbalance in the normal reward processing homeostasis [35, 36]. At the molecular level, Acetylcholine (ACh) from the cholinergic interneurons of the NAc binds to neuronal nicotinic acetylcholine receptors (nAChR), and modulates the release of dopamine (DA) and reinforced behaviours [37]. Interestingly, sucrose has been shown, albeit indirectly, to affect the release of DA in the NAc via nAChRs [38], suggesting that the nAChRs are a promising target for pharmacotherapy.

While numerous nAChR subtypes have been identified in the limbic system, including the NAc, identification of the nAChR subtype(s) involved in mediating and maintaining sucrose consumption is not known. Varenicline, a partial agonist at α4β2*, α6β2*, and α3β2*-nAChRs (*denotes the presence of other possible subunits in the receptor complex) and a full agonist at α7 and α3β4* subtypes [39, 40] reduces nicotine cravings and withdrawal symptoms [41] as well as in reducing alcohol consumption [42]. Varenicline displays efficacy for smoking cessation by firstly, moderately enhancing DA release in the NAc and secondly, attenuating nicotine-induced DA release by competitively blocking the nAChR binding site [43, 44]. Given the involvement of acetylcholine in appetite, it will be interesting to test the efficacy of varenicline in reducing sucrose consumption In addition, testing of other nAChR drugs may help to identify the potential nAChR subunits being targeted.

## 2. Materials and Methods

### 2.1 Drugs

5% (w/v) sucrose and 0.2% (w/v) saccharin solutions (Sigma, ST. Louis, USA) were prepared in RO-tap water. Varenicline (6,7,8,9-tetrahydro-6,10-methano-6*H* pyrazino[2,3-*h*][3]benzazepine tartrate), mecamylamine (*N*,2,3,3-Tetramethylbicyclo[2.2.1]heptan-2-amine hydrochloride), and (-)-cytisine ((1*R*,5*S*)-1,2,3,4,5,6-hexahydro-1,5-methano-8*H*-pyrido[1,2-*a*][1,5]diazocin-8-one) were purchased from Tocris (Bristol, UK).

### 2.2 Animals and Housing

Five-week-old male Wistar rats (183g ± 14g) (ARC, WA, Australia), were individually housed in ventilated dual level Plexiglas cages. The rats were acclimatized to the individual housing conditions, handling, and reverse-light cycle 5 days before the start of the experiments. All rats were housed in a climate-controlled 12-h reversed light/dark cycle (lights off at 9 a.m.) room with unlimited access to food (standard rat chow) and water. The experimental procedures followed the ARRIVE guidelines and were approved by the Ethics Committees of the Queensland University of Technology Animal Ethics Committee and the University of Queensland Animal Ethics Committee, in accordance with the European legislation (European Communities Council Directive of 24 November 1986, 86/609/EEC).

### 2.3 Intermittent-access two-bottle choice drinking paradigm

The intermittent access 5% sucrose two-bottle choice drinking paradigm was adapted from [45]. All fluids were presented in 300-ml graduated plastic bottles with stainless-steel drinking spouts inserted through two grommets in the front of the cage following the commencement of the dark light cycle. Two bottles were presented simultaneously: one bottle containing water; the second bottle containing 5% (w/v) sucrose. The placement of the 5% (w/v) sucrose bottle was alternated with each exposure to control for side preferences. Bottles were weighed 30 min, 2 h, and 24 h after the fluids were presented, and measurements were taken to the nearest 0.1gram. The weight of each rat was also measured to calculate the grams of sucrose intake per kilogram of body weight. On the Monday after the end of the housing acclimatization period, rats (183 ± 14 g, n = 10–12) were given access to one bottle of 5% (w/v) sucrose and one bottle of water. After 24 h, the sucrose bottle was replaced with a second water bottle that was available for the next 24 h. This pattern was repeated on Wednesdays and Fridays; All other days the rats had unlimited access to water. Drug administration began after the rats had maintained stable baseline drinking levels (20 ± 5 g/kg) of the 5% (w/v) sucrose solution for (a) short-term exposure [~4 weeks (13 drinking sessions)]; and, (b) long-term exposure [~12 weeks (37 drinking sessions)]. The mean body weight at the start of drug testing was 373 ± 26g for short-term, and 550 ± 48 g for long-term. The nAChR agonists, antagonists, and vehicle were administered as described.

In order to compare voluntary baseline sucrose consumption in animals using the intermittent-access protocol versus the continuous access protocol, a separate group (n  =  10) of 5-week old wistar rats was maintained on a continuous-access 5% sucrose protocol for 4 weeks. These rats were given access to one bottle of 5% sucrose and one bottle of water 24 hours a day, seven days a week for the duration of the experiment. Sucrose and water bottles were weighed everyday (total of 56 sessions with the bottles weighed) to calculate sucrose intake and preference. Animal weights were also recorded on these days. The placement of the sucrose bottle was alternated each day to control for side preferences.

Furthermore, to determine the effect of varenicline on the consumption of a non-caloric sweetener, saccharin 0.2% (w/v), was presented to a separate group of rats (n = 10) as per the intermittent-access protocol described herein. 4 weeks from the start of saccharin consumption, rats were administered varenicline using a latin square at doses as described. Lastly, a separate group of rats on the sucrose intermittent-access protocol that were designated for autoradiography were killed by decapitation and the brains quickly removed, frozen in isopentane on dry ice and stored at -80°C. The brains were then sectioned (8 μm) at the level of the striatum using a cryostat (Leica Microsystems Inc., Deerfield, IL) set at -15 to -20°C. The sections were thaw mounted onto poly-L-lysine coated slides, dried and stored at -80°C until used for autoradiography. Rats consuming water (i.e. no sucrose) were used as control.

### 2.4 Treatment schedules

Wistar rats were divided into groups of 10–12. For rats on short-term drinking and also long-term drinking, Varenicline (vehicle, 0.3, 1 and 2 mg/kg) was administered to each animal using a Latin square design. Furthermore, in a group of rats (n = 8), food consumption after administration of varenicline was recorded to the nearest 0.1 gram at all time points. Subsequently, after return to baseline drinking, mecamylamine (vehicle, 0.5, 1 and 2 mg/kg), was administered as before. In a separate group of rats, (-)-cytisine (vehicle, 2 and 4 mg/kg) was administered using the Latin square design. Lastly, a separate group of rats drinking saccharin short-term was administered varenicline as before. As per Latin square design, each rat served as its own control. The doses used in this study reflect those used in the extant literature [46–51].

All drugs were dissolved in saline and administered as a subcutaneous (s.c.) injection, in a volume of 1 ml/kg, 30 min before sucrose and water bottles were presented. All drug solutions were prepared immediately before each injection.

### 2.5 ^125^I-Epibatidine Autoradiography

Binding of ^125^I-epibatidine (2200 Ci/mmol; Perkin Elmer Life Sciences, Boston, MA, USA) was done as previously reported [52]. Slides were pre-incubated at 22°C for 15 min in buffer containing 50 mM Tris, pH 7.5, 120 mM NaCl, 5 mM KCl, 2.5 mM CaCl_2_, and 1.0 mM MgCl_2_. They were incubated for 40 min with 0.015 nM ^125^I-epibatidine in the presence or absence of α-conotoxin MII (α-CtxMII) (100 nM). They were then washed, dried, and exposed to Kodak MR Film with ^125^I-microscale standards (GE Healthcare, Chalfont St. Giles, Buckinghamshire, UK) for 5–7 days. Nonspecific binding was assessed in the presence of 100 μM nicotine and was similar to the film blank.

### 2.6 Dopamine transporter autoradiography

Binding to the dopamine transporter (DAT) was measured using ^125^I-RTI-121 (2200 Ci/mmol; Perkin Elmer Life Sciences, Boston, MA, USA), as previously described [53]. Thawed sections were pre-incubated twice for 15 min each at 22°C in 50 mM Tris-HCl, pH 7.4, 120 mM NaCl, and 5 mM KCl, and then incubated for 2 h in buffer with 0.025% bovine serum albumin, 1 μM fluoxetine, and 50 pM ^125^I-RTI-121. Fluoxetine was used to block off-target binding to the serotonin transporters Sections were washed at 0°C for 4 × 15 min each in buffer and once in ice-cold water, air dried, and exposed for 2 day to Kodak MR film with ^125^I-microscale standards (GE Healthcare). Nomifensine (100 μM) was used to define non-specific binding.

### 2.7 Data Analyses

The ImageQuant program from GE Healthcare was used to determine the optical density values from autoradiographic films. Background tissue values were subtracted from total tissue binding to evaluate specific binding of the radioligands. Specific binding values were then converted to fmol/mg tissue using standard curves determined from ^125^I standards. Care was taken to ensure that sample optical density readings were within the linear range.

All statistics and curve fittings were conducted using GraphPad Prism 6 (Graph Pad Software Co., San Diego, CA, USA). Statistical comparisons were performed using unpaired t-test analysis, one-way analysis of variance (ANOVA) followed by a Newman—Keuls multiple comparisons test or two-way ANOVA followed by Bonferroni post hoc test. A value of p ≤0.05 was considered significant. All values are expressed as the mean ± SEM of the indicated number of animals, with release values for each animal representing the average of 6–15 signals from 1–2 slices.

## 3. Results

### 3.1 Varenicline reduces sucrose consumption using the intermittent-access two-bottle choice paradigm

**Fig 1:**
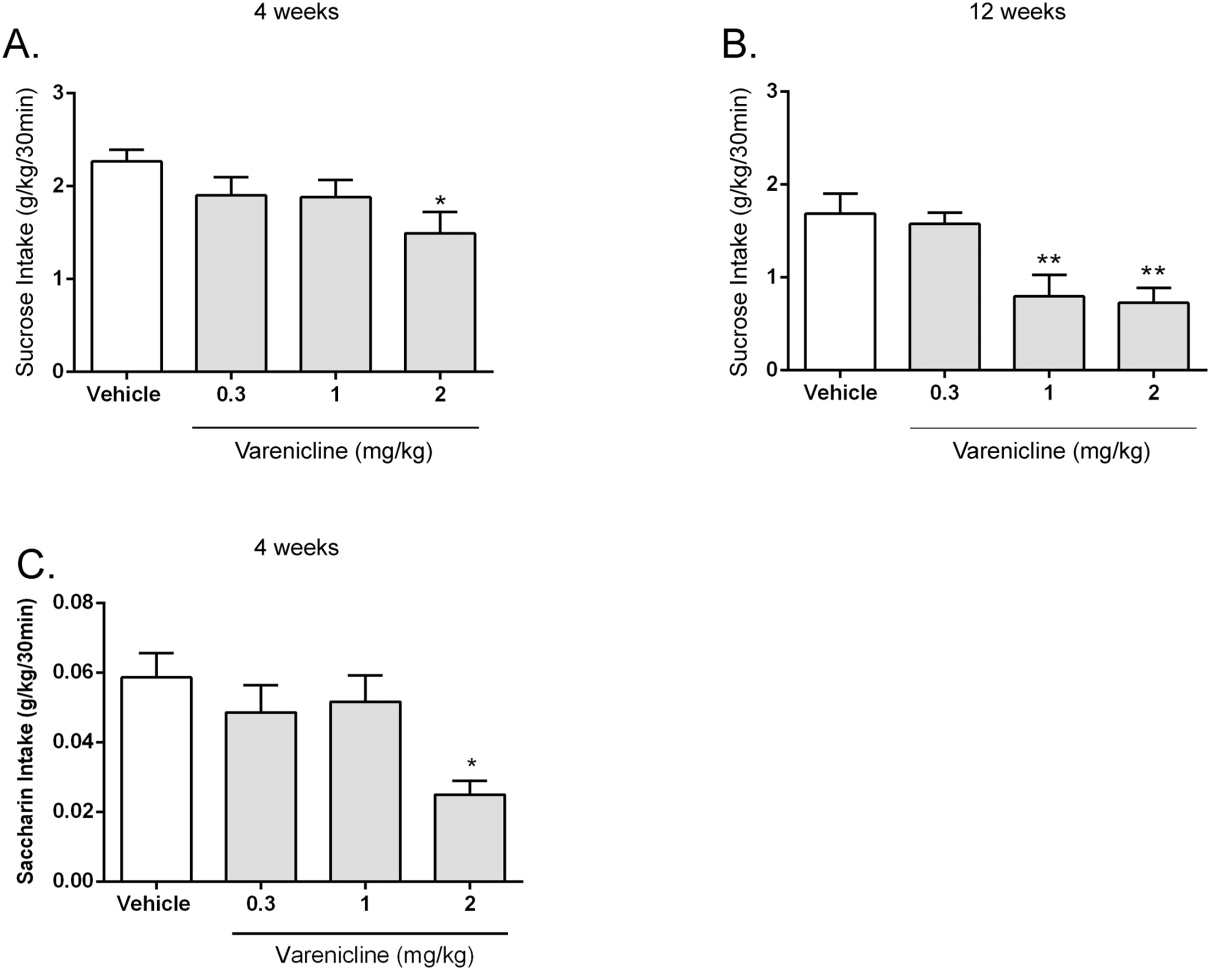
Long-term exposure to sucrose (12 weeks) in rats using the intermittent-access two-bottle choice paradigm increased the efficacy of varenicline. Varenicline (2 mg/kg) significantly decreased sucrose consumption (Fig 1A) after short-term (4 weeks) exposure to sucrose. Whereas, both (1 and 2 mg/kg of varenicline significantly decreased sucrose consumption (Fig 1*B*) following long-term (12 weeks) sucrose exposure. Varenicline (2 mg/kg) significantly decreased saccharin consumption (Fig 1C) after short-term (4 weeks) exposure to saccharin. The values are expressed as mean sucrose intake (g/kg) ± SEM (repeated-measures ANOVA followed by Newman—Keuls post hoc test). *, *P* < 0.05; **, *P* < 0.01 compared with vehicle, *n* = 10–12.

To examine the effects of varenicline in short-term (4 week) and long-term (12 week) sucrose-consuming rats, we used the intermittent-access two-bottle choice drinking paradigm [54]. Sub-cutaneous (s.c) administration of varenicline in rats consuming sucrose short-term (Fig 1A) decreased sucrose intake [*F* (3, 33) = 3.8, *P* < 0.05]. Post hoc analysis revealed that only 2 mg/kg significantly decreased sucrose consumption. In contrast, in long-term sucrose drinking rats (Fig 1B), while varenicline decreased sucrose consumption [*F* (3, 24) = 15.24, *P* < 0.0001], post hoc analysis revealed both 1 and 2 mg/kg significantly decreased sucrose consumption in a dose-dependent manner compared with vehicle. Also, systemic varenicline did not affect chow consumption at any of the tested timepoints and all effective doses, both short-term and long-term. Interestingly, s.c administration of varenicline in rats consuming saccharin short-term (4 weeks) (Fig 1C) decreased saccharin intake [*F* (3, 24) = 5.67, *P* < 0.05]. Post hoc analysis revealed that only 2 mg/kg significantly decreased saccharin consumption. In all cases above, significance was observed at the 30 min timepoint, with no significance at 2hr and 24hr timepoint.

Furthermore, in contrast to the effect of varenicline on sucrose and saccharin consumption in short-term (4 weeks) sucrose-consuming animals on the intermittent-access protocol, varenicline did not decrease sucrose consumption in animals on continuous-access to sucrose short-term (4 weeks) (Data not shown). It is to be noted that rats on intermittent-access consumed significantly more sucrose in the first 30 mins of bottle presentation than rats on continuous-access as determined by an unpaired two-tailed t-test (t = 4.025, df = 13, *P* < 0.01). Hence, all further experiments in this study utilised the intermittent-access protocol. In all cases, water consumption was not affected.

### 3.2 Mecamylamine, a non-competitive, non-selective nAChR antagonist reduces sucrose consumption using the intermittent-access two-bottle choice paradigm

**Fig 2:**
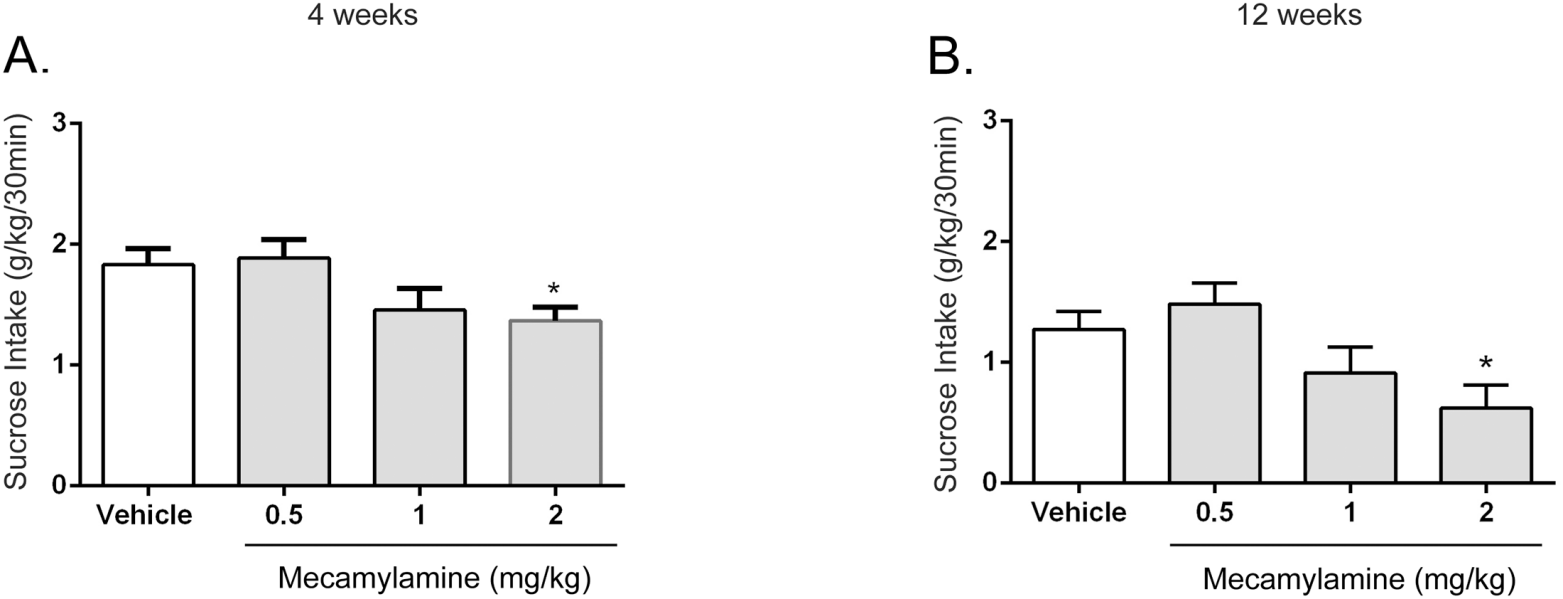
Mecamylamine significantly decreased sucrose intake in rats consuming sucrose short-term (4 weeks) and long-term (12 weeks) using the intermittent-access two-bottle choice paradigm. Mecamylamine (2 mg/kg) significantly decreased sucrose consumption in short-term (4 weeks) and long-term (12 weeks) sucrose exposure rats (Fig 2A and 2B). The values are expressed as mean sucrose consumed (g/kg) ± SEM (repeated-measures ANOVA followed by Newman—Keuls post hoc test). *, *P* < 0.05; **, *P* < 0.01; ***, *P* < 0.001 compared with vehicle, *n* = 12.

We next examined the effect of mecamylamine, a non-competitive, non-selective nAChR antagonist, on sucrose consumption in the same intermittent-access two-bottle choice paradigm as stated above. Mecamylamine decreased sucrose consumption in short-term [*F* (3, 33) = 5.9, *P* < 0.01 30min; *F* (3, 33) = 10.91, *P* <0.001 2hr] and long-term sucrose-consuming rats [*F* (3, 21) = 4.6, *P* < 0.05 30 min; *F* (3, 21) = 10.42, *P* <0.001 2hr]. Post hoc analysis revealed that the dose of 2 mg/kg significantly decreased sucrose consumption at the 30 min timepoint in short-term (Fig 2A) and long-term sucrose-consuming rats (Fig 2B), and at the 2hr timepoint as well. Also, 1 mg/kg was significant short-term at the 2hr timepoint. Sucrose consumption was not affected at the 24hr timepoint for the doses tested. Water consumption was not affected at any timepoint and dose.

### 3.3 Cytisine reduces sucrose consumption using the intermittent-access two-bottle choice paradigm

**Fig 3:**
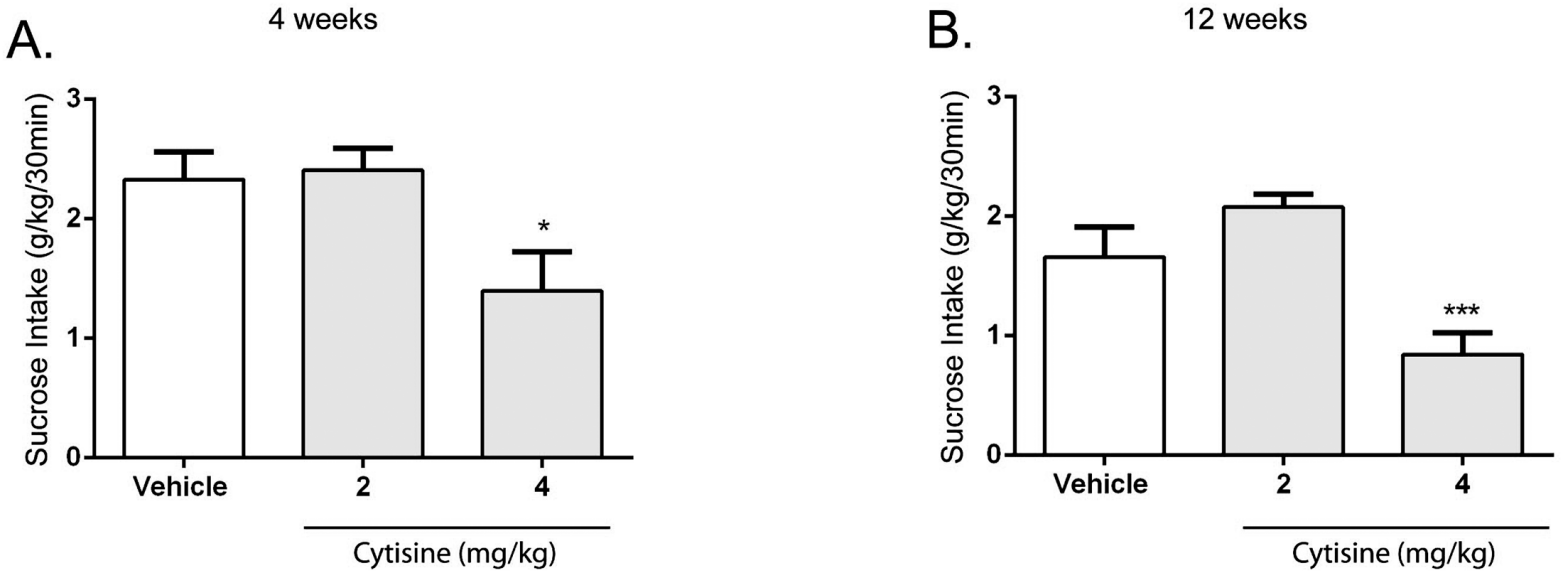
Cytisine significantly decreased sucrose intake in rats consuming sucrose short-term (4 weeks) and long-term (12 weeks) using the intermittent-access two-bottle choice paradigm. Cytisine (4 mg/kg) significantly decreased sucrose consumption (Fig 3A and 3B) after the onset of drinking in short-term (4 weeks) and long-term (12 weeks) sucrose exposure rats. The values are expressed as mean sucrose consumed (g/kg) ± SEM (repeated-measures ANOVA followed by Newman—Keuls post hoc test). *, *P* < 0.05; **, *P* < 0.01; ***, *P* < 0.001 compared with vehicle, *n* = 12.

A second group of rats were tested with (-)-cytisine, a β2-selective nAChR agonist. Cytisine significantly decreased sucrose consumption in short term [*F* (2, 22) = 7.18, *P* < 0.01 30min; *F* (2, 22) = 6.82, *P* <0.01 2hr] and long-term sucrose-consuming rats [*F* (2,20) = 19.43, *P* < 0.0001 30min; *F* (2,20) = 12.94, *P* < 0.001 2hr). Post hoc analysis revealed that the dose of 4 mg/kg significantly decreased sucrose consumption at the 30 min timepoint in short-term (Fig 3A) and long-term sucrose-consuming rats (Fig 3B), and at the 2hr timepoint as well. Sucrose consumption was not affected at the 24hr timepoint for the doses tested. Also, water consumption was not affected at any timepoint and dose.

### 3.4 Exposure to both short-term (4 week) and long-term (12 week) sucrose consumption increases α4β2* and decreases α6β2* nAChR subtype binding in the nucleus accumbens

**Fig 4:**
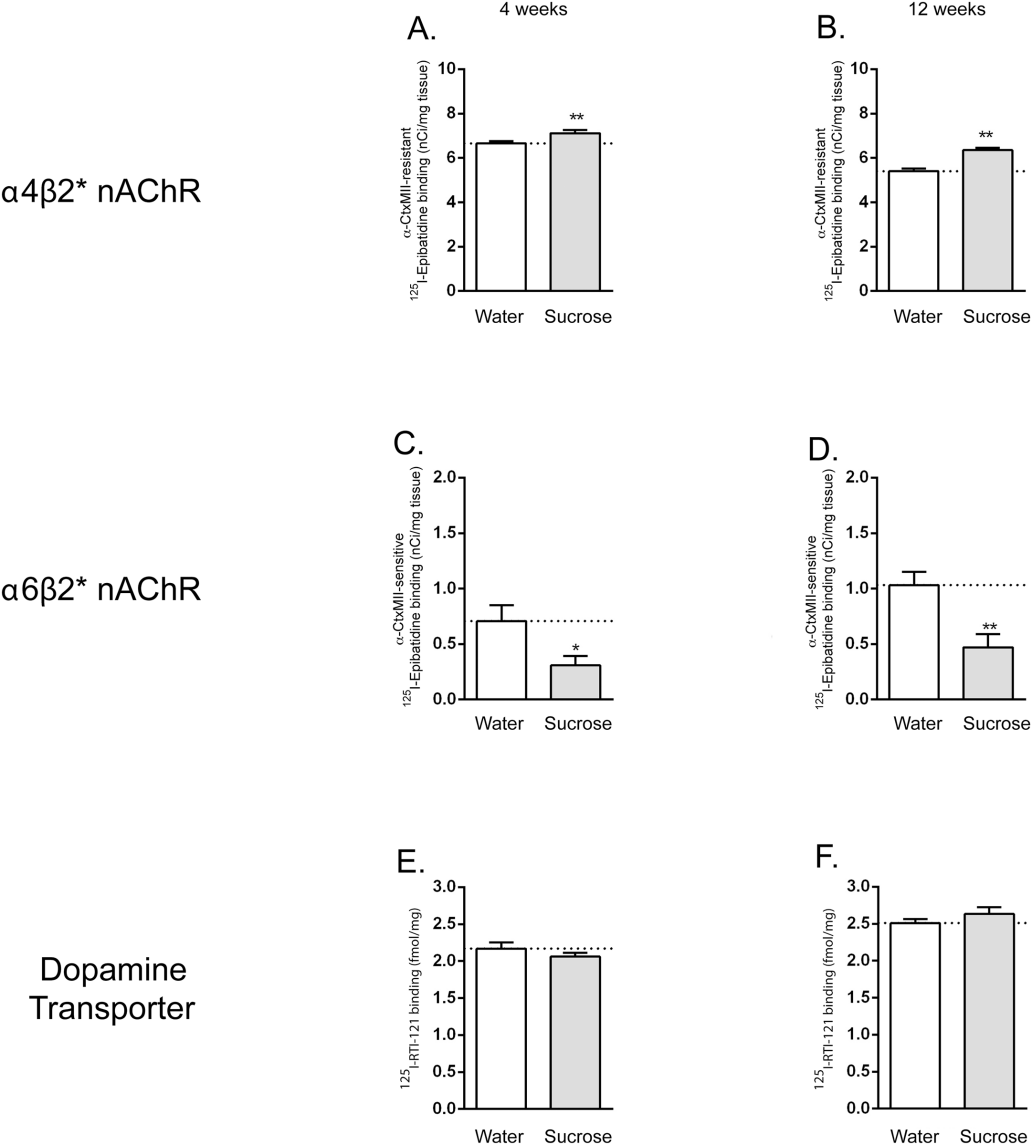
Long-term sucrose intake (12 weeks) increases α4(nonα6)β2* nAChR and decreases α6β2* nAChR levels in rat nucleus accumbens (NAc). Quantitative analyses of α4(nonα6)β2* nAChR binding using ^125^I-Epibatidine binding in the absence and presence of α-CtxMII show show a significant increase in α4(nonα6)β2* nAChRs (A and B) with a decrease in α6β2* nAChRs (C and D) after short-term (4 week) and long-term (12 week) sucrose exposure in the intermittent-access two-bottle choice paradigm. Dopamine transporter (DAT) as determined by ^125^I-RTI-121 binding does not show any significant change short-term (4 week) and long-term (12 week) (E and F respectively). Each value represents the mean _ SEM of four animals per group. Significance of difference from vehicle-treated rats, ****p<0.0001, **p < 0.01, *p < 0.05.

The striatum contains two major nAChRs populations, the α4β2* and α6β2* subtypes [55]. To determine how long-term sucrose treatment modified α4β2* and α6β2* modulated subtype expression in the brain, we measured ^125^I-epibatidine binding in the absence and presence of α-CtxMII, which blocks α6β2* nAChRs (Fig 4A and 4B). Binding determined in the presence of α-CtxMII represents that occurring at α4β2* nAChRs, while the difference between total and α4β2* nAChR binding is defined as α6β2* nAChR binding. α4(non α6)β2* nAChRs were significantly increased in the NAc of both short-term and long-term sucrose-treated animals (unpaired T-test; p = 0.024 and <0.0001, respectively). By contrast, α6β2* nAChRs (Fig 4C and 4D) were significantly decreased short-term (unpaired t-test; p = 0.028) as well as long-term (unpaired t-test; p = 0.0035) with sucrose treatment. Lastly, we also compared the binding of the dopamine transporter (DAT) by ^125^I-RTI-121 binding to assess the modulation of dopamine shuttling in sucrose treated rats. There was no significant change observed short-term (4 week) and long-term (12 week) (unpaired T-test; p = 0.290 and 0.263, respectively).

## 4. Discussion

The present study shows that systemic administration of varenicline produced a dose-dependent reduction of sucrose consumption using the intermittent-access two-bottle choice paradigm, especially after long-term sucrose consumption. It is known that varenicline, a partial agonist at neuronal α4β2*, α6β2*, and α3β2*-nAChRs and a full agonist at α7 and α3β4* nAChR subtypes [39, 40], reduces nicotine cravings and withdrawal symptoms [41], as well as attenuates ethanol consumption in animal studies [42]. Furthermore, varenicline has been shown to mediate its effect at the level of the NAc [56], a key region of the limbic reward pathway in the brain. It has previously been shown that feeding to satiety increases ACh in the accumbens [57], specifically in the context of sucrose consumption [58]. Interestingly, it is the dysregulation of balance between dopamine (DA) and acetylcholine (ACh) in the limbic system, especially in the NAc that has been found to drive and maintain behaviours that perpetuate addiction to substances of abuse [59, 60]. Interestingly, varenicline did not affect sucrose consumption in the short-term continuous-access two-bottle choice paradigm suggesting that intermittent access to sucrose may contribute to neurological changes for which varenicline is effective. Future studies, however, will be necessary to ascertain this. Furthermore, it is particularly interesting that varenicline decreased not only sucrose but also saccharin consumption without affecting water intake, suggesting palatability to sweet foods as important, especially in terms of the possible involvement of the limbic system. Furthermore, following longer (12 weeks) exposure to sucrose, a lower dose of varenicline was as effective in reducing sucrose consumption as the higher dose. This differential response could be attributed to the changes observed in binding for the α4β2 containing nAChR subunits as demonstrated in this study.

We also observed that mecamylamine, a non-selective non-competitive nAChR antagonist reduced sucrose consumption. Our finding is supported by a recent study which found that mecamylamine decreased pavlovian incentive motivation for sugar [61] and operant self-administration, albeit at much higher doses [62]. Further, an *in vitro* application of mecamylamine in the NAc, decreased ghrelin-mediated accumbal DA release [63]. Cytisine, a β2 selective nAChR agonist, marketed as a smoking cessation aid Tabex in eastern European countries, also reduced sucrose consumption. An earlier report, however, investigating the effects of cytisine on ethanol consumption concluded that cytisine (3 mg/kg, s.c) did not reduce voluntary sucrose intake [64]. In addition to the potential species differences [65], there were many procedural differences between our experiments and those reported by Sajja and Rahman (2011). Most notably, Sajja and Rahman (2011) used a lower highest dose (3 mg/kg) versus 4 mg/kg in our study. However, if these factors may be attributable to the observed differences is unclear at present.

Furthermore, It is to be noted that the effect of mecamylamine and cytisine on reducing sucrose consumption for a longer time period in our study (2hr vs 30min), maybe due to the broader range of nAChR subunits targeted by mecamylamine and cytisine as compared to those targeted by varenicline [66, 67]. Furthermore, differential pharmacokinetics of mecamylamine and cytisine as compared to varenicline, may also contribute to this observed effect. These possibilities however are speculative and will need to be investigated in future studies. Also, nausea or locomotor effects may be ruled out because the doses used in our study for varenicline (0.3–2 mg/kg), mecamylamine (0.5–2 mg/kg) and cytisine (2–4 mg/kg) are similar to the doses used in previous studies, namely varenicline (0.3–3 mg/kg), mecamylamine (0.5–4 mg/kg) and cytisine (0.3–5 mg/kg) [46–51, 68–70].

The observation that not only the partial agonists varenicline and cytisine, but also the antagonist mecamylamine, reduced sucrose consumption may provide insight into the molecular mechanism whereby β2* nAChR drugs induce their effect. One possible interpretation is that it involves nAChR desensitization. Although it is quite well established that acetylcholine and nAChR agonists initially lead to nAChR activation, this is quickly followed by molecular modifications that lead to channel closing and a receptor block or desensitization [71–73]. It has been suggested that nicotine and nicotinic receptor drugs exert their overall behavioural effects via desensitization of nicotinic receptors has been suggested to underlie their mechanism of action, at least in part, on analgesia, depression, smoking cessation and others [74–76]. If nAChR agonists exert their beneficial effects via a receptor blockade, antagonists may be more useful from a clinical standpoint. Alternatively, partial nAChR agonists, such as varenicline, may be more effective therapeutically.

In the current study, we also found that long-term sucrose exposure resulted in an increase in α4β2* and a decrease in α6β2* nAChR receptors in the NAc. Interestingly, administration of nicotine results in similar changes in the α4β2* and α6β2* nAChRs levels, and to a similar magnitude as that obtained in the present study with sucrose [77–79]. Although the mechanisms responsible for this are still not fully understood, it has been suggested that changes in α4β2* and α6β2* nAChRs contribute to nicotine re-enforcement and self-administration [80–84]. By analogy, the observed changes in nAChRs with sucrose intake may underlie the addictive properties of sucrose. It is to be noted that at present it is unclear if the observed changes in the levels of α4β2* and α6β2* nAChRs are due to the palatability of sucrose or due to increased caloric intake. Whilst varenicline had similar effects on saccharin and sucrose consumption in our study, suggesting palatability as an attractive proposition, future studies are warranted to exclude increased caloric intake as a putative causative factor for the observed changes in nAChR expression levels. This will also help to clarify the mechanism underlying the receptor changes presented in our study. In terms of sugar consumption and, more generally, food consumption, speculation remains regarding the addictive properties of these foods. Indeed, a recent review by Hebebrand and colleagues [85] discerns the nuanced difference between food addiction and the much preferred nomenclature of eating addiction. Despite these speculations, the behavioural and neural correlates in relation to sugar consumption, posit the mesolimbic pathway as an attractive target for pharmacotherapy intervention.

In conclusion, pharmacological interference with nAChRs affects sucrose consumption. Furthermore, based on the various nAChRs agonists and antagonists tested, we conclude that β2* nAChRs are involved in mediating pharmacological effects on sucrose consumption. We demonstrate that sucrose mediates an increase in α4β2* and decrease in α6β2* nAChRs in the NAc, suggesting this region as a highly plausible candidate in modulating sucrose consumption. Further studies are warranted to validate the putative role of the NAc in modulating sucrose consuming behaviour as a function of the nAChRs. Lastly, our study suggests a completely novel putative treatment strategy for reducing sugar consumption.

## Supporting Information

S1 Table Standard-chow consumption on treatment with varenicline. (DOCX)
