# Supplementary material for: Neuronal Nicotinic Acetylcholine Receptor Modulators Reduce Sugar Intake
Source: PLoS One. 2016 Mar 30;11(3):e0150270. doi: 10.1371/journal.pone.0150270 (PMC4814119; doi:10.1371/journal.pone.0150270)
Supplement: S1 Table — (DOCX) [file pone.0150270.s001.docx]

**Table S1. Chow consumption represented as grams (g) chow per gram body weight (gBW) (at 30 min post presentation of 5% sucrose solution)**

| **(A) Short-term:** | |  | |
| --- | --- | --- | --- |
|  | Standard chow consumption (g/30min/gBW) (x 10^-4^) | | |
| Animal No. | Vehicle | | Varenicline 2 mg/kg |
| 1 | \| 114.79 \| \| --- \| | | 132.48 |
| 2 | 116.67 | | 123.91 |
| 3 | 142.86 | | 129.66 |
| 4 | 126.58 | | 138.96 |
| 5 | 106.53 | | 98.34 |
| 6 | 135.57 | | 112.42 |
| 7 | 120.17 | | 109.46 |
| 8 | 110.47 | | 109.75 |
| **Avg (± SEM)** | \| **121.70** \| \| --- \| \| **± 4.42** \| | | **119.37**  **± 4.94** |

P= 0.65

| **(B) Long-term:** | |  | |  | |
| --- | --- | --- | --- | --- | --- |
|  | Standard chow consumption (g/30min/gBW) (x 10^-4^) | | | | |
| Animal No. | Vehicle | | Varenicline 1 mg/kg | | Varenicline 2 mg/kg |
| 1 | 101.92 | | 85.88 | | 103.68 |
| 2 | 130.77 | | 112.77 | | 100.31 |
| 3 | 112.34 | | 91.25 | | 81.13 |
| 4 | 95.53 | | 93.58 | | 108.20 |
| 5 | 92.02 | | 112.25 | | 85.91 |
| 6 | 84.64 | | 102.38 | | 96.50 |
| 7 | 109.20 | | 97.97 | | 118.61 |
| 8 | 87.30 | | 95.79 | | 105.52 |
| **Avg (± SEM)** | **101.72**  **± 5.42** | | **98.98**  **± 3.41** | | **99.98**  **± 4.28** |

P= 0.9 F (7, 14) = 0.09792
